# Systematic review of survival following liver or lung metastasectomy for metastatic anal squamous cell carcinoma

**DOI:** 10.1308/rcsann.2023.0005

**Published:** 2024-03-18

**Authors:** L Hurt, E Barlow, M Davies, DA Harris, C Barrington, RL Harries

**Affiliations:** Swansea Bay University Health Board, UK

**Keywords:** Metastatic anal squamous cell carcinoma; Liver metastases; Systematic review; Metastasectomy

## Abstract

**Introduction:**

Metastatic anal squamous cell carcinoma (SCC) carries a poor prognosis and the evidence base for surgical resection of metastases remains limited. The aim of this study was to establish the survival outcomes for patients undergoing metastasectomy for anal SCC.

**Methods:**

A systematic review was performed using the MEDLINE^®^, Embase^®^, Cochrane and PubMed^®^ databases. Studies were considered for inclusion in the review if they involved patients aged >18 years with a diagnosis of stage IV anal SCC who underwent metastasectomy for liver and/or lung metastases. The primary outcome measure was overall survival. Secondary outcome measures were disease free survival, early morbidity according to the Clavien–Dindo classification and quality of life, measured using a validated scoring tool. Risk of bias was assessed with the ROBINS-I (Risk Of Bias In Non-randomised Studies – of Interventions) tool.

**Results:**

There were 10 studies with a total of 98 patients. There was heterogeneity in results reporting, with recurrence free survival the most reported outcome. For all studies reporting on liver metastasectomy, the one-year overall survival rate was 87%. In studies with adequate follow-up reported, the three and five-year overall survival rates were 53% and 38% respectively. Only one study reported on lung metastasectomy patients; the overall median survival was 24 months. None of the studies reported on quality of life measures. The ROBINS-I tool identified a critical risk of bias in six studies, a serious risk in one study and a moderate risk in three studies.

**Conclusions:**

The evidence base for metastasectomy in metastatic anal SCC is limited. Further information is required to inform future treatment methods and use of a standardised outcomes reporting method is needed to support this.

## Introduction

In the UK, anal cancer is relatively uncommon, comprising less than 1% of new cancer diagnoses annually.^1^ The incidence has been showing an increasing trend globally, where risk factors include human papillomavirus (HPV) and human immunodeficiency virus (HIV) infection, immunosuppression and smoking.^2^ The mainstay of treatment for non-metastatic anal squamous cell carcinoma (SCC) is chemotherapy and radiotherapy, with one-year and five-year survival rates of 84.8% and 58.7% respectively.^1^ However, the prognosis for those with metastatic stage IV disease is poor, with a one-year survival rate of only 53%. In the UK, treatment for metastatic disease usually involves systemic chemotherapy or best supportive care. The role of surgical resection in liver or lung metastases is still debated and the evidence base remains limited. The aim of this systematic review was to establish the survival outcomes for patients undergoing liver and/or lung metastasectomy for anal SCC.

## Methods

This systematic review was undertaken in accordance with the PRISMA (Preferred Reporting Items for Systematic reviews and Meta-Analyses) guidelines.^3^ It was registered prospectively with the PROSPERO database (CRD42021242289).

A systematic search of the MEDLINE^®^, Embase^®^, Cochrane and PubMed^®^ databases was performed for papers published up until 10 September 2021. The search terms employed were: “liver*”, “hepatic”, “exp liver”, “exp liver tumor”, “lung”, “pulmonary”, “exp lung tumor”, “exp lung”, “resect*”, “lobectom*”, “hepatectom*”, “metastesectom*”, “pneumonectom*”, “exp liver resection”, “exp lung resection”, “exp metastasis resection”, “metastasis”, “metast*”, “stage IV”, “stage 4”, “advanced adj3 metast*”, “oligometast*”, “anal”, “anus”, “exp anal canal”, “exp anus or exp anus carcinoma/or exp anus cancer”, “squamous”, “exp squamous cell”, “carcinoma”, “cancer”, “tumor or tumour or neoplas*”, “exp carcinoma”, “squamous cell carcinoma”. These were used alone or in combination. The “relevant articles” function was utilised to broaden the search. A manual search of reference lists of eligible studies was also performed.

### Inclusion and exclusion criteria

Studies that were eligible for inclusion in the analysis were randomised controlled trials, prospective observational studies, retrospective cohort series and case reports while meta-analyses, systematic reviews, comments/letters or conference abstracts were excluded. Only studies of patients aged >18 years with a diagnosis of stage IV SCC of the anus who underwent metastasectomy for either liver and/or lung metastases were eligible. Those investigating surgical resection in comparison with other treatment modalities were included provided data were extractable for outcomes relating to patients undergoing metastasectomy. Any studies involving patients with metastatic SCC with a variety of primary sites where data specific to anal origin were not extractable were excluded from this systematic review.

### Outcome measures

The primary outcome measure was overall survival (date of treatment to date of death or most recent follow-up). Secondary outcome measures were disease free survival (date of surgery to date of diagnosis of recurrence or most recent follow-up), early morbidity according to the Clavien–Dindo classification^4^ and quality of life as measured using a validated scoring tool.

### Data extraction

Study selection and data extraction was performed by two reviewers independently (LH and EB), with disagreements being solved by discussion or through the involvement of a third independent reviewer (RLH). Studies were selected in a two-step process. Initially, titles were screened for inclusion, followed by an overview of the abstract. Following this, studies that were selected had their full text evaluated. If deemed eligible for inclusion, data were then extracted. Demographic data included study information such as names of authors and year of publication, study design, number of patients, age, sex, site of metastases, surgical intervention, other metastasis directed treatment modalities (e.g. chemotherapy or radiotherapy) and HIV status. Outcomes data including overall survival and mortality rates, disease specific survival and mortality rates, recurrence, early morbidity and quality of life were recorded.

### Risk of bias

The ROBINS-I (Risk Of Bias In Non-randomised Studies – of Interventions) assessment tool was used to assess risk of bias.^5^

### Statistical analysis

Analysis was planned to be descriptive. It was predicted that multivariate analysis would not be required.

## Results

**Figure 1:**
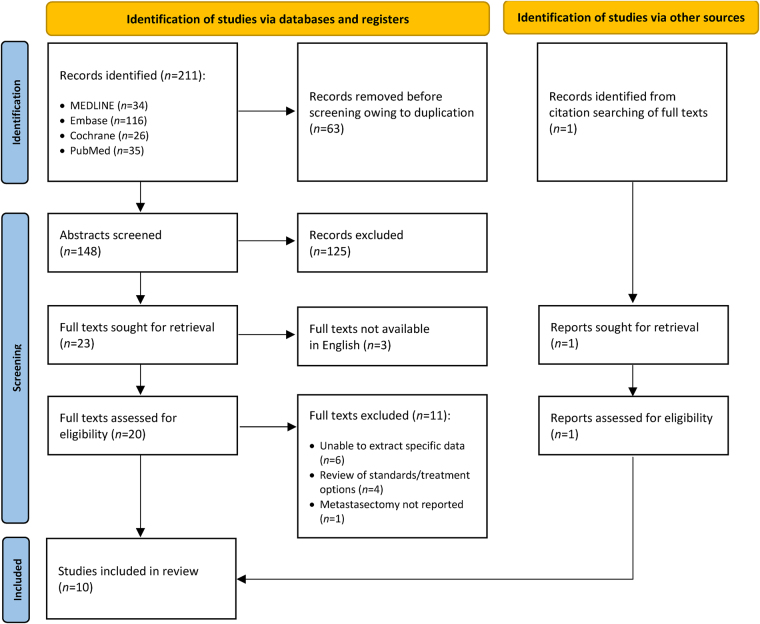
Flowchart of study selection

A total of 148 studies were initially identified and screened for inclusion. Of these, 23 were assessed for eligibility and 10 were included in this review (Figure 1 and Table 1).^6–15^ Reasons for exclusion included inability to extract data specific to liver or lung metastasis arising from anal SCC and inability to extract anal SCC specific outcomes.

**Table 1:** Summary of outcomes for included studies

Study	Study design	Number of patients	Site of metastasis	Mean number of metastases	Median size of largest metastasis	Patients with metastases present at initial diagnosis	Overall survival rates	Median overall survival*	Median recurrence free survival*	Median length of follow-up*	Postoperative complications
Tanum, 1991^6^	Case series	2	Liver	NR	NR	NR	NR	NR	24 months	NR	NR
Gurfinkel, 2005^7^	Case report	1	Liver	2	NR	1	NR	Alive at 36 months^†^	Recurrence free at 36 months^†^	36 months^†^	Uncomplicated
Tokar, 2006^8^	Case report	1	Liver	2	6cm × 6cm	1	NR	Alive at 5 months	24 months	5 months	Uncomplicated
Pawlik, 2007^9^	Prospective multicentre cohort analysis	27	Liver	1	5.8cm	9	1-year: 74.4% 3-year: 27.7% 5-year: 22.9%	22.3 months	9.6 months	17.9 months	NR
Gassman, 2012^10^	Case report	1	Liver	1	6.1cm	1	NR	9 months	3 months	NR	Uncomplicated
Lupinacci, 2013^11^	Case report	1	Liver	3	NR	1	NR	Alive at 7 months	Recurrence free at 7 months	7 months	Uncomplicated
Gnanajothy, 2016^12^	Case report	1	Liver	1	2.5cm	1	NR	Alive at 19 months	Recurrence free at 19 months	19 months	NR
Sousa, 2016^13^	Case report	1	Liver	3	13.1cm × 10.7cm × 10.6cm	0	NR	Alive at 13 months	Recurrence free at 13 months	13 months	Uncomplicated
Sclafani, 2018^14^	Case series	7	Liver (*n*=4) Lung (*n*=3)	2	3.1cm	NR	NR	37 months, 25 months	7 months, 4 months	21 months, 19 months	NR
Engstrand, 2021^15^	Retrospective multicentre cohort analysis	56	Liver	2	3.1cm	16	1-year: 92.6% 3-year: 59.5% 5-year: 45.4%	49.7 months	15.9 months	22 months	Minor: 19^‡^ Major: 4^§^

NR = not reported or unable to extract specific data

*From date of first organ directed therapy or surgical resection

^†^From date of diagnosis rather than surgery

^‡^Clavien–Dindo grade I–IIIa

^§^Clavien–Dindo grade IIIb–IV

In the 10 included studies, there were 98 patients (27 male, 71 female) with a median age range of 52–67 years across the studies. HIV and HPV status were poorly reported; one case series^14^ and three case reports^11–13^ outlined HPV and/or HIV status. One patient with a solitary liver metastasis in a case series was known to be HIV positive^14^ and one patient with one liver metastasis in a case report was HPV positive.^12^ Resection as a standalone treatment was the most common metastasis directed therapy, seen in 75 patients across 4 studies.^6,9,14,15^ Eleven patients (from 7 studies) had chemotherapy and resection: five had neoadjuvant chemotherapy,^7,10–13^ three had adjuvant chemotherapy,^14^ and three had combined neoadjuvant and adjuvant chemotherapy.^8,14^ As well as surgical resection, eight patients underwent ablation,^9,15^ and one patient underwent both chemotherapy and radiotherapy to the hepatic tumour bed.^14^

There was heterogeneity in results reporting, with the most reported outcome measure being recurrence free survival. Two multicentre analyses reported on one-year, three-year and five-year overall survival. Pawlik *et al* reported the one, three and five-year survival rates as 74.4%, 27.7% and 22.9% respectively^9^ whereas Engstand *et al* reported these as 92.6%, 59.50% and 45.4% respectively.^15^ Across all studies for liver metastasectomy, the one-year overall survival rate was found to be 87% (93 patients).^6–15^ In studies with adequate follow-up reported, the three-year overall survival rate was 53% (89 patients)^6–9,14,15^ and the five-year survival rate was 38% (83 patients).^8,9,14,15^ For lung metastasectomy patients, the only study reported an overall median survival of 24 months.^14^ The median overall survival for individual studies is reported in Table 1.

Engstrand *et al* found a median recurrence free survival of 15.9 months with a median follow-up period of 22 months (56 patients).^15^ Pawlik *et al* reported a median recurrence free survival of 9.6 months with a median follow-up period of 17.9 months (27 patients).^9^ For lung metastasectomy, the only case series reported the median recurrence free survival as four months.^14^ The median recurrence free survival for individual studies is reported in Table 1.

Five studies reported an uncomplicated postoperative recovery following metastasectomy. Engstrand *et al* reported complications according to the Clavien–Dindo classification: 19 patients suffered minor complications (Clavien–Dindo grades I–IIIA) and 4 had major complications following surgery (Clavien–Dindo grades IIIB–IV).^15^ None of the studies included in this review reported on quality of life measures.

The ROBINS-I assessment tool^5^ identified six studies where the overall risk of bias was critical while for one study, the risk was serious and for three studies, it was moderate (Table 2). Both of the cohort studies were found to have a moderate risk of bias.

**Table 2:** Risk of bias assessment according to the ROBINS-I tool^6^

Study	Baseline confounding	Selection of participants	Classification of interventions	Deviation from intended interventions	Missing data	Measurement of outcomes	Selection of reported results	Overall risk of bias
Tanum, 1991^6^	Serious	Critical	Moderate	NR	Low	Serious	Moderate	Serious
Gurfinkel, 2005^7^	Critical	Critical	Moderate	NR	Low	Critical	Moderate	Critical
Tokar, 2006^8^	Critical	Critical	Moderate	NR	Low	Critical	Moderate	Critical
Pawlik, 2007^9^	Serious	Low	Moderate	NR	Low	Moderate	Low	Moderate
Gassman, 2012^10^	Critical	Critical	Moderate	NR	Low	Critical	Moderate	Critical
Lupinacci, 2013^11^	Critical	Critical	Moderate	NR	Low	Critical	Moderate	Critical
Gnanajothy, 2016^12^	Critical	Critical	Moderate	NR	Low	Critical	Moderate	Critical
Sousa, 2016^13^	Critical	Critical	Moderate	NR	Low	Critical	Moderate	Critical
Sclafani, 2018^14^	Serious	Low	Moderate	NR	Low	Moderate	Moderate	Moderate
Engstrand, 2021^15^	Moderate	Serious	Serious	NR	Low	Moderate	Low	Moderate
NR = not reported								

## Discussion

A systematic review in 2018 found that liver metastasectomy for gastric cancer has a significant impact on overall survival compared with medical therapy alone.^16^ This has also been found in breast cancer^17^ and favourable outcomes have been identified in cases of colorectal cancer.^18^

The UK’s Department of Health and Social Care has published an action plan to improve diagnosis and treatment of rare conditions including anal SCC.^19^ One of the underpinning themes in the report is mapping the rare disease research landscape to identify gaps and priorities for future funding. Our systematic review adds to the existing body of evidence aimed at exploring novel approaches to disease management and highlights the gaps in that evidence base.

Approximately 10–20% of patients with anal SCC will develop distant metastases.^20,21^ Distant metastases are widely recognised as difficult to treat, with the mainstay of treatment in the UK being systemic chemotherapy or best supportive care. However, with up to 10% of anal SCC patients presenting with distant metastases in the absence of local recurrence or failure, it is a logical step to explore options for surgical resection. Overall, there is limited evidence for survival outcomes following liver or lung resection of anal SCC metastasis. Both national and international guidelines relating to the management of anal SCC metastatic disease have been unable to make recommendations on the role of surgical resection, with recommendations for systemic chemotherapy or best supportive care only.^22–25^ The Association of Coloproctology of Great Britain and Ireland guidance states: “There is no systematically reviewed evidence to recommend resection or ablation of oligometastases.”^24^

In this systematic review, there were no randomised studies and the studies with the highest level of evidence were two multicentre cohort studies,^9,15^ only one of which was prospective. Most of the included studies were case reports and case series with a small number of patients. This means that there is a high risk of bias overall, with the two multicentre analyses identified as having a moderate risk of bias (Table 2).

Overall survival was reported at one, three and five years for both multicentre cohort studies. Improved survival was reported in the most recent study.^15^ This could be explained by medical advances as these studies are 14 years apart. Reported recurrence rates across all studies showed poor disease free survival. There appeared to be few short-term major complications reported although it is recognised that the evidence base is limited and likely to have a reporting bias. Unfortunately, no studies included quality of life measures. It is therefore not possible to determine whether patients enjoyed an improved (perhaps less symptomatic) quality of life following metastasectomy and there are no data to determine the role of surgical resection as a symptom alleviating palliative procedure.

Although metastasectomy appears to be an acceptable and safe treatment option with few major short-term complications reported, the small number of included patients in these studies makes establishing meaningful survival outcomes difficult. The survival rates reported in this paper highlight the need for clinicians to counsel patients carefully on expected outcomes following diagnosis of liver or lung metastases. When considering surgical resection, patients must be aware of the expected prognosis so that they can make a fully informed decision after weighing up the risks of metastasectomy (including postoperative recovery) and the benefits that it may offer. It is important to note that this review is non-comparative and purely descriptive owing to the nature of the studies included; it is not feasible to undertake a true comparison between non-operated patients and those who underwent metastasectomy.

What is not known from the included studies is the role of different multimodal treatments (chemotherapy and/or immunotherapy) in combination with surgical resection and its effect on outcomes. The current evidence base for chemotherapy and immunotherapy as treatment without metastasectomy shows equally poor overall survival. The InterAACT study, an international multicentre randomised controlled trial on advanced anal cancer, included 91 patients assigned to either carboplatin plus paclitaxel or cisplatin plus 5-fluorouracil (5-FU).^26^ The median overall survival was 12.3 months for cisplatin plus 5-FU (95% confidence interval [CI]: 9.2–17.7 months) compared with 20 months (95% CI: 12.7 months – not reached) for carboplatin plus paclitaxel (hazard ratio: 2.00, 95% CI: 1.15–3.47, *p*=0.014). The KEYNOTE-028 study found a median overall survival of 9.3 months with pembrolizumab^27^ and the NCI9673 study found a median overall survival of 11.5 months with nivolumab in patients who had received prior systemic therapy for advanced disease.^28^

It should be noted, however, that the characteristics of patients included in trials for medical therapies will differ from those in trials undergoing surgical resection, as discussed in this review. This will likely contribute to the poorer outcomes reported in the trials for chemotherapy and/or immunotherapy compared with those reported in this paper for patients who underwent metastasectomy. The better outcome for surgical resection may partly be explained by careful patient selection (for example, selecting patients with low tumour burden).

Further research would be useful to ascertain current patient outcomes, with homogenous results reporting to determine the efficacy of liver or lung resection in metastatic anal SCC, in combination with multimodal treatments, focusing on outcomes such as recurrence free survival, overall survival, morbidity and mortality with quality of life measures included. We would recommend that studies follow the core outcomes set for clinical trials of chemoradiotherapy interventions for anal cancer and extend this to interventions for metastatic presentations.^29^ The current UK data collection for the National Bowel Cancer Audit excludes anal cancer.^30^ One method of increasing patient numbers is the mASCARA (multinational Anal Squamous Cell CArcinoma Registry and Audit) study, which is collecting international collaborative retrospective and prospective data on patients with an anal SCC diagnosis.^31^ This study hopes to provide answers presently lacking in the available literature.

## Conclusions

This systematic review has brought together the available literature on outcomes after surgical resection of anal SCC liver and lung metastases. It has highlighted likely implications following resection although it is limited by low levels of evidence and an overall low number of patients. There is an urgent need for improvement in the reporting of outcomes in these patients to establish the optimal treatment options.
